# HnRNPA1 interacts with G-quadruplex in the *TRA2B* promoter and stimulates its transcription in human colon cancer cells

**DOI:** 10.1038/s41598-019-46659-x

**Published:** 2019-07-16

**Authors:** Tatsuya Nishikawa, Yuki Kuwano, Yumiko Takahara, Kensei Nishida, Kazuhito Rokutan

**Affiliations:** 1 Department of Pathophysiology, Institute of Biomedical Sciences, Tokushima University Graduate School, Tokushima, 770–8503 Japan; 2 Student Lab, Tokushima University Faculty of Medicine, Tokushima, Japan

**Keywords:** Oncogenes, Gene regulation

## Abstract

The human *TRA2B* gene consists of 10 exons and 9 introns and produces 5 splice isoforms (*TRA2β1* to *TRA2β5*). *TRA2B* exon 2 encodes multiple premature termination codons. *TRA2β1* lacks exon 2 and is translated into a functional transformer 2β (Tra2*β*) protein, whereas *TRA2β4* contains 10 exons and works as a functional RNA. Overexpressed Tra2*β* and ectopic expression of *TRA2β4* may be oncogenic. We found that heterogeneous nuclear ribonucleoprotein (hnRNP)A1 and hnRNPU interacted with *TRA2β4* exon 2. Minigene assays revealed that hnRNPA1 facilitated inclusion of exon 2, whereas hnRNPU promoted its skipping. However, knockdown of hnRNPA1 or hnRNPU reduced both *TRA2β1* and *TRA2β4* levels, and overexpression of these hnRNPs increased levels of both isoforms, suggesting that hnRNPA1 and hnRNPU mainly regulate the transcription of *TRA2B*. In fact, hnRNPA1 and hnRNPU positively regulated the promoter activity of *TRA2B*. Circular dichroism analyses, electrophoretic mobility shift assays and chromatin immunoprecipitation assays demonstrated the presence of G-quadruplex (G4) formation in the promoter of *TRA2B*. Formation of G4 suppressed *TRA2B* transcription, whereas hnRNPA1, but not hnRNPU, interacted with the G4 to facilitate transcription. Our results suggest that hnRNPA1 may modulate *TRA2B* transcription through its regulation of G4 formation in its promoter in colon cancer cells.

## Introduction

The human *TRA2B* gene encodes transformer 2β (Tra2*β*) protein, a member of the serine/arginine-rich splicing factor-like family. Tra2β regulates alternative splicing of several genes such as calcitonin/calcitonin gene-related peptide (*CGRP*), myosin phosphatase targeting subunit 1 (*MYPT1*), survival motor neuron (*SMN*) and microtubule-associated protein *tau* (*TAU*) in a concentration-dependent manner^1–4^. Tra2β is overexpressed in breast, cervical, ovarian, prostate and lung cancers^5–9^. Tra2β levels were greater in high-grade gliomas than low-grade gliomas, and Tra2β knockdown resulted in suppression of cell proliferation and inhibition of cell migration^10^. In human prostate cancer, overexpression of Tra2β was a significant predictor of recurrence and poor survival^6^. Thus, *TRA2B* has been recognized as an oncogene.

The human *TRA2B* gene consists of 10 exons and 9 introns and generates 5 mRNA isoforms (*TRA2β1 to TRA2β5*) through alternative splicing and usage of alternative promoters or polyadenylation sites^11^. Among them, *TRA2β1* and *TRA2β4* are the main isoforms^12^. *TRA2β1* mRNA consists of exons 1–10 except for exon 2, whereas *TRA2β4* isoform contains all 10 exon^13^. Although *TRA2β1* mRNA is translated into Tra2β protein, *TRA2β4* does not encode full-length protein because of the presence of premature stop codons in exon 2. We reported that oxidative stress stimulated *TRA2B* transcription through heat shock factor 1^14^ and increased *TRA2β4* production through HuR^13^. *TRA2β4* is preferentially expressed in the nuclei of human colon cancer cells through association with nucleolin^15^, facilitating abnormal cell growth^12^. Tra2β protein interacts with the *BCL2* 3′-UTR and promotes abnormal growth of colon cancer cells^16^. Based on these findings, both Tra2β and *TRA2β4* are considered to be new targets for treatment of colon cancer. However, the transcriptional and post-transcriptional regulation of *TRA2B* are not fully understood.

In the present study, we identified heterogeneous nuclear ribonucleoprotein (hnRNP) A1 and hnRNPU as *TRA2β4*-binding proteins using biotinylated RNA pull-down and RNA immunoprecipitation assays. The hnRNP family functions as major splicing factors as well as transcriptional regulators for various genes^17^. In fact, both hnRNPA1 and hnRNPU could upregulate the promoter activity of *TRA2B*. However, we found that hnRNPA1, but not hnRNPU, interacted with a G-quadruplex (G4) structure in the *TRA2B* promoter. G4 reportedly has various functions related to transcription^18,19^, replication^20,21^, telomere maintenance^22,23^, DNA damage repair^24^, posttranscriptional regulation^25^ and many others. Notably, recent studies have revealed that G4 resides in the promoters of many oncogenes or tumor suppression genes^26^. In particular, the transcription of *KRAS*^27^ or *C-MYC*^28^ was reported to be regulated via G4 in their promoter regions. Here, we suggest that hnRNPA1 may regulate *TRA2B* transcription through interaction with G4 in its promoter region.

## Results

### Effects of knockdown or overexpression of hnRNPA1 or hnRNPU on *TRA2β1* and *TRA2β4* expression

**Figure 1:**
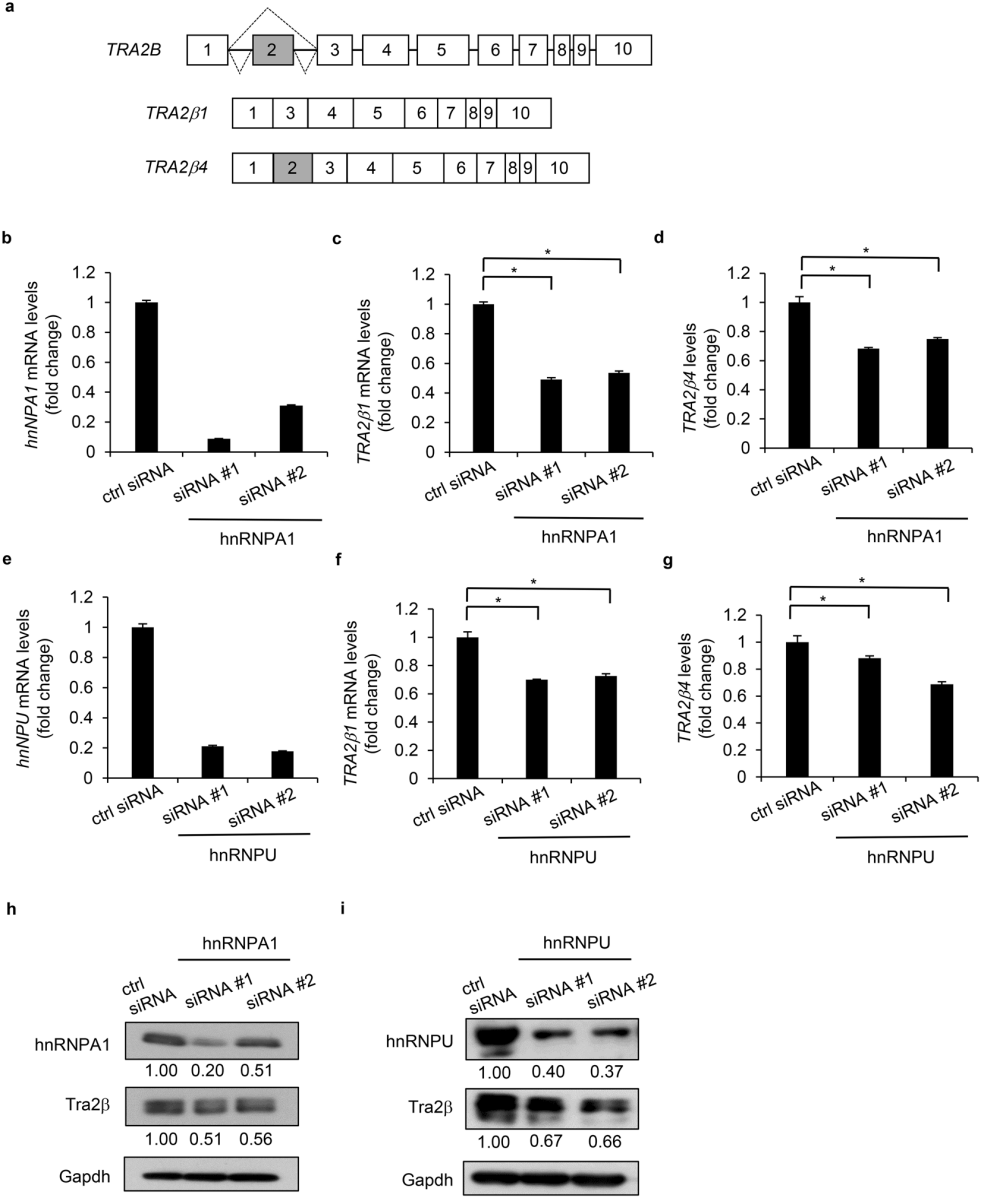
Effects of knockdown of hnRNPA1 or hnRNPU on *TRA2B* expression. (**a**) Schematic diagrams of *TRA2B*, *TRA2β1* and *TRA2β4*. Exons are indicated by Arabic numbers in the boxes. Dotted lines show alternative splicing of exon 2. (**b**) After HCT116 cells were treated with 20 nM control (ctl) siRNA or one of the 2 *hnRNPA1* siRNAs (#1 and #2) for 48 h, *hnRNPA1* mRNA levels were measured by RT-qPCR using *GAPDH* mRNA as an internal control for normalization. Values are expressed as relative changes, compared with those in control siRNA-treated cells (means ± SD, n = 4). (**c**,**d**) After knockdown of *hnRNPA1*, *TRA2β1* and *TRA2β4* levels were measured by RT-qPCR and compared with those in control siRNA-treated cells. Values are means ± SD, n = 4. (**e**) After treatment of HCT116 cells with 20 nM control siRNA or one of the 2 *hnRNPU* siRNAs (#1 and #2) for 48 h, *hnRNPU* mRNA levels were measured by RT-qPCR. The relative values are means ± SD, n = 3. (**f**,**g**) Changes in the expression levels of *TRA2β1* and *TRA2β4* after hnRNPU knockdown. (**h**) After *hnRNPA1* knockdown, hnRNPA1 and Tra2β levels were measured by Western blot analysis using GAPDH as a loading control. Immunoreactive signals of corresponding bands were quantified by densitometry. Relative values (means ± SD, n = 4) are indicated below each figure. (**i**) Changes in hnRNPU and Tra2β proteins after *hnRNPU* knockdown were measured by Western blot analysis. Values are means ± SD, n = 4. *Significantly different, compared with control siRNA or mock (*p* < 0.05 by two-tailed Student’s *t*-test).

First, we prepared 3 cDNA probes (full-length *TRA2β1*, full-length *TRA2β4*, and *TRA2β4* exon 2) and used them to identify *TRA2β4*-binding proteins through exon 2 (Fig. 1a). After biotinylated RNA pull-down assays using each probe, purified proteins were resolved by sodium dodecyl sulfate-polyacrylamide gel electrophoresis (SDS-PAGE), transferred to a polyvinylidene fluoride membrane, and visualized by silver staining. As shown in Supplementary Fig. S1a, 2 protein bands with molecular masses of ~100 kDa and ~40 kDa preferentially bound to exon 2 and exon 2-containing *TRA2β4*. As described in a previous study^15^, hnRNPU was present in the 100-kDa proteins. Liquid chromatography/mass spectrometry (LC-MS/MS) was employed to identify hnRNPA1 as one of the 40 kDa proteins (Supplementary Fig. S1a). We were particularly interested in hnRNPA1 and hnRNPU as exon 2-binding proteins, since these 2 RNA-binding proteins are known to be associated with accelerated cell growth^29–31^. Additional biotinylated RNA pull-down assays (Supplementary Fig. S1b) and RNA immunoprecipitation assays (Supplementary Fig. S1c,d) demonstrated that both hnRNPA1 and hnRNPU were preferentially associated with *TRA2β4*, compared with *TRA2β1* or *GAPDH*.

Next, we evaluated how hnRNPA1 and hnRNPU modified the expression of *TRA2β1* and *TRA2β4*. After knockdown of hnRNPA1 or hnRNPU, real-time quantitative PCR **(**RT-qPCR) was employed to measure *TRA2β1* and *TRA2β4* using primer sets that had been designed to specifically amplify these 2 transcripts^13^. To avoid off-target effects, we used 2 commercially available sets of siRNAs for each target protein. All of the siRNAs effectively reduced their target transcripts (Fig. 1b,e). Unexpectedly, silencing of hnRNPA1 or hnRNPU significantly decreased the levels of both *TRA2β1* and *TRA2β4* (Fig. 1c,d,f,g). Tra2β protein levels were also reduced after knockdown of hnRNPA1 or hnRNPU (Fig. 1h,i).

**Figure 2:**
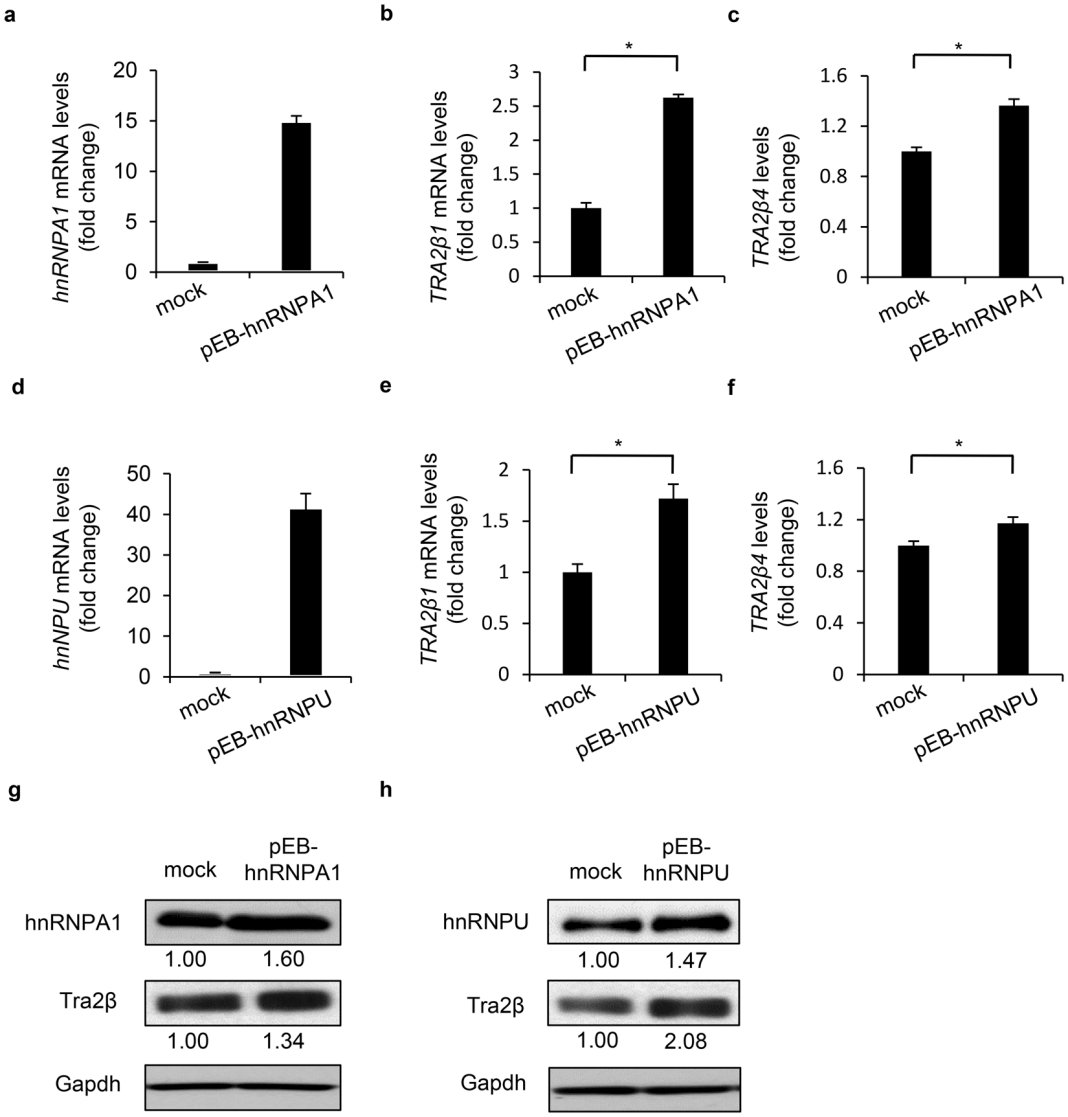
Effects of transient overexpression of *hnRNPA1* or *hnRNPU* on *TRA2B* expression. (**a**) Full-length *hnRNPA1* mRNA was prepared and cloned into a pEBMulti vector. HCT116 cells were transfected with this vector for 48 h. The amounts of *hnRNPA1* mRNA were measured by RT-qPCR. Fold-changes were calculated by comparing with the values of the empty vector-transfected cells (means ± SD, n = 4). (**b**,**c**) Levels of *TRA2β1* and *TRA2β4* were measured by RT-qPCR in HCT116 overexpressing *hnRNPA1* or transfected with an empty vector. *GAPDH* mRNA was used as an internal control. Values are expressed as fold-changes compared with those in the empty vector-transfected cells (means ± SD, n = 4). *Significantly different by two-tailed Student’s *t*-test (*p* < 0.05). (**d**) After transfection with the pEBMulti vector carrying full-length *hnRNPU* mRNA or an empty vector, *HnRNPU* mRNA levels in the cells were measured by RT-qPCR. Values are means ± SD, n = 4. (**e**,**f**) *TRA2β1* and *TRA2β4* levels in the hnRNPU-overexpressing cells were measured by RT-qPCR and compared with those in the empty vector-transfected, control cells. Values are means ± SD, n = 4. *Significantly different by two-tailed Student’s *t*-test (*p* < 0.05). (**g**,**h**) Tra2β, hnRNPA1, and hnRNPU in hnRNPA1- or hnRNPU-overexpressing cells and control cells were measured by Western blot analysis.

We also examined the effects of transient overexpression of hnRNPA1 or hnRNPU using the pEBMulti vector system. Overexpressed mRNA and protein levels of hnRNPA1 or hnRNPU are shown in Fig. 2a,d,g,h. Densitometric analysis showed that hnRNPA1 and hnRNPU increased 1.60 ± 0.12 and 1.47 ± 0.13-fold, respectively (mean ± SD, n = 4). The overexpressed hnRNPA1 and hnRNPU significantly increased not only *TRA2β1* but also *TRA2β4* levels (Fig. 2b,c,e,f) in association with increases in Tra2β protein levels (Fig. 2g,h). Thus, both hnRNPA1 and hnRNPU seemed to positively regulate *TRA2B* transcription.

### Roles of hnRNPA1 and hnRNPU in alternative splicing of *TRA2β* pre-mRNA

*TRA2β4* exon 2-binding proteins, hnRNPA1 and hnRNPU, are thought to regulate *TRA2β* pre-mRNA processing. To test this, we employed minigene assays. As shown in Fig. 1, siRNAs #1 and #2 for hnRNPA1 or hnRNPU showed similar knockdown efficacies. Therefore, only siRNA #1 for hnRNPA1 or hnRNPU was used in the following experiments. The minigene contains the entire sequence from *TRA2B* exon 1 to exon 3 in a pcDNA3.1 vector (Supplementary Fig. S2a). After HCT116 cells were treated with hnRNPA1, hnRNPU or control siRNA for 48 h, an empty vector (mock) or a vector containing the *TRA2B* minigene was transfected into the cells. After reverse transcription using a gene-specific primer, transcribed products were amplified by PCR and separated by agarose gel electrophoresis.

Treatment with hnRNPA1 siRNAs decreased *TRA2β1* by 50% (Fig. 1c) and *TRA2β4* by 30% (Fig. 1d), whereas hnRNPA1 siRNA decreased the *TRA2β4*/*TRA2β1* ratio by 20% compared with control siRNA treatment (Supplementary Fig. S2b), suggesting that hnRNPA1 may facilitate inclusion of exon 2. Treatment with hnRNPU siRNAs decreased both *TRA2β1* and *TRA2β4* by 20 to 30% (Fig. 1f,g), whereas hnRNPU siRNA increased the *TRA2β4*/*TRA2β1* ratio over 3-fold (Supplementary Fig. S2c), indicating that hnRNPU may facilitate skipping of exon 2. Thus, although hnRNPA1 and hnRNPU could modify the alternative splicing of *TRA2B* exon 2, the effects were minimal when compared to the effects on the transcriptional regulation of *TRA2B*.

We also evaluated whether the interaction of hnRNPA1 or hnRNPU affected the turnover of *TRA2β1* and *TRA2β4*. We found that knockdown of hnRNPA1 or hnRNPU did not change the stability of *TRA2β1* and *TRA2β4* (Supplementary Fig. S3).

### Interaction between hnRNPA1 and G-quadruplex in the *TRA2B* promoter

**Figure 3:**
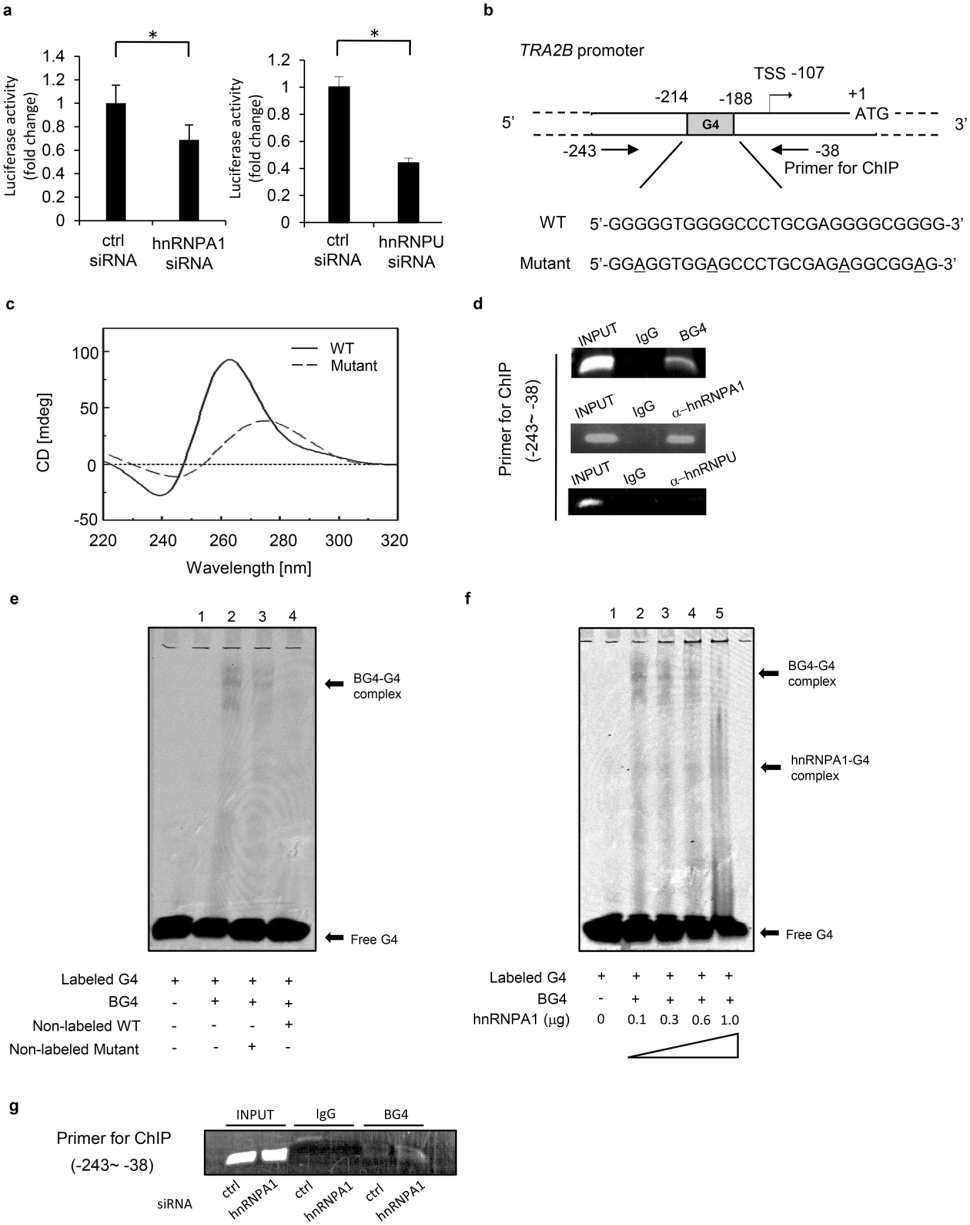
G-quadruplex (G4) formation in the *TRA2B* promoter and its interactions with hnRNPA1. (**a**) After treatment of HCT116 cells with control, *hnRNPA1*, or *hnRNPU* siRNA for 24 h, the pGL-3 luciferase construct with the *TRA2B* proximal promoter (from −398 to +107 bp) was co-transfected with pRL-CMV vector for 24 h. The dual-luciferase reporter assay was employed in *hnRNPA1* knockdown cells (left), and in *hnRNPU* knockdown cells (right). (**b**) Mutations of the putative G4 sequence in the *TRA2B* promoter are underlined. (**c**) Circular dichroism (CD) spectra were obtained from the synthesized G4 nucleotide (WT, wild type) and its mutant. (**d**) Chromatin immunoprecipitation (ChIP)-PCR analysis was performed using a G4-containing promoter fragment (−231 to −68 bp) with anti-BG4, anti-hnRNPA1 and anti-hnRNPU antibodies. (**e**) Electrophoretic mobility shift assay (EMSA) was done using an IRDye700-labeled G4 sequence and BG4 antibody. (**f**) IRDye700-labeled G4 sequence and BG4 antibody was incubated with purified hnRNPA1 protein and then analyzed by EMSA. These results were confirmed by 3 independent experiments and representative data are shown. (**g**) ChIP-PCR assay with the same primer set as (**d**) to compare cells treated with control or hnRNPA1 siRNA.

To reveal how hnRNPA1 regulated the transcription of *TRA2B*, we first assessed the promoter activity of *TRA2B* using a dual-luciferase reporter assay. We previously characterized the basal promoter region (−166 to −21 bp) of *TRA2B*^14^. The transcription start site is located 107 bp upstream from the translational start site. HCT116 cells were transfected with the pGL3 basic vector containing −398 to +107 bp of the 5′-flanking region of *TRA2B* and used for assessment of the *TRA2B* promoter activity. Knockdown of hnRNPA1 resulted in a small but significant decrease of the luciferase activity (Fig. 3a). Those results were consistent with the finding that knockdown of hnRNPA1 reduced the levels of both *TRA2β1* (Fig. 1c) and *TRA2β4* (Fig. 1d).

To elucidate the mechanism for hnRNPA1-dependent regulation of promoter activity, we were particularly interested in the presence of a latent G-quadruplex (G4) sequence (5′-GGGGGTGGGGCCCTGCGAGGGGCGGGG-3′) in the *TRA2B* gene promoter region (−214 to −188 bp) (Fig. 3b). Circular dichroism analysis indicated that this sequence had a unique spectrum with the highest peak at 265 nm and the lowest peak at 240 nm, results characteristic of a parallel G4 structure. Introduction of the mutation (5′-GGAGGTGGAGCCCTGCGAGAGGCGGAG-3′) abolished the peaks characteristic of the G4 structure (Fig. 3c). To confirm the formation of the G4 structure, we also employed electrophoretic mobility shift assays (EMSA) using an IRDye700-labeled G4 sequence and a G4 specific antibody (BG4). EMSA showed a shifted G4-BG4 complex band that disappeared after adding an excess amount of non-labeled wild-type G4, but not mutant G4 nucleotides (Fig. 3e). To further validate the formation of the G4 structure, we employed chromatin immunoprecipitation (ChIP)-PCR assays. As shown in Fig. 3d, BG4 bound to a chromatin region that included the G4 sequence of the *TRA2B* promoter. Moreover, hnRNPA1 associated with the same region (Fig. 3d). Considering these results, hnRNPA1 likely interacted with the G4 sequence to promote *TRA2B* gene transcription. To confirm this hypothesis, we further demonstrated the involvement of hnRNPA1 in G4 formation of the *TRA2B* promoter. As shown in Fig. 3f, the BG4-G4 complex was abolished by addition of purified hnRNPA1 protein, suggesting that G4 formation was inhibited by hnRNPA1. In addition, ChIP assay with BG4 antibody showed that the G4 in the *TRA2B* promoter increased in hnRNPA1 knockdown cells compared to control cells (Fig. 3g). These results might suggest that hnRNPA1 bound to G4 sequence in the *TRA2B* promoter and unfolded its G4 structure.

**Figure 4:**
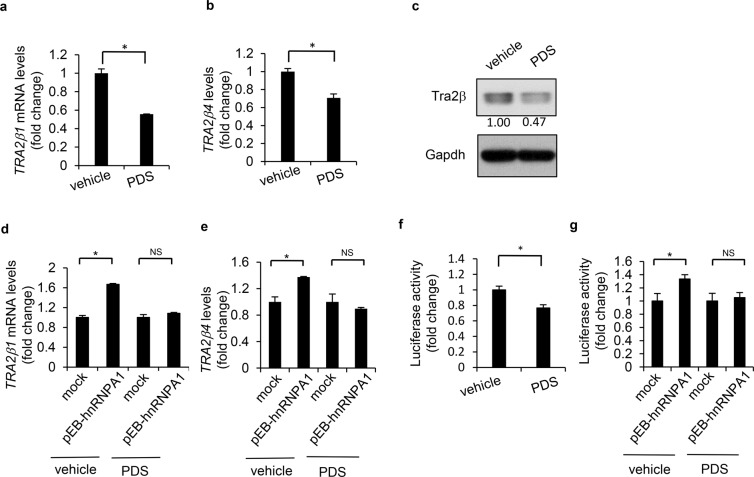
Changes in *TRA2B* transcription by Pyridostatin treatment. (**a**) and (**b**) After a 24 h treatment of HCT116 cells with 20 μM pyridostatin (PDS), a G4 stabilizer, *TRA2β1* and *TRA2β4* levels were measured by RT-qPCR. Values (fold-changes) are means ± SD, n = 4. *Significantly different by two-tailed Student’s *t*-test (*p* < 0.05). (**c**) Tra2*β* levels in PDS-treated cells were measured by Western blot analysis. (**d**,**e**) HCT116 cells were transfected with the pEBMulti vector carrying full-length *hnRNPA1* or empty vector (mock). After these cells were treated with 20 μM PDS for 24 h, *TRA2β1* and *TRA2β4* levels were measured by RT-qPCR. Values (fold-changes) are means ± SD, n = 4. *Significantly different by two-tailed Student’s *t*-test (*p* < 0.05). (**f**,**g**) After PDS treatment of untreated, *hnRNPA1*-overexpressing or mock-transfected HCT 116 cells, the *TRA2B* promoter activity was measured by a dual-luciferase reporter assay. Values (fold-changes) are means ± SD, n = 4. *Significantly different by two-tailed Student’s *t*-test (*p* < 0.05).

For further analysis of interaction between hnRNPA1 and G4 in the *TRA2B* promoter, we treated HCT116 cells with a well-known G4 stabilizer, pyridostatin (PDS). Treatment with PDS decreased the mRNA levels of *TRA2β1* (Fig. 4a) and *TRA2β4* (Fig. 4b), and Tra2β protein (Fig. 4c). Furthermore, overexpression of hnRNPA1 significantly increased both *TRA2β1* and *TRA2β4* (Fig. 2b,c). In contrast, these increases were completely blocked by treatment with PDS (Fig. 4d,e). In accordance with these findings, PDS treatment decreased the promoter activity (Fig. 4f), suggesting that the G4 structure may play a significant role in regulating transcription of *TRA2B*. PDS treatment blocked the increase of promoter activity by hnRNPA1 overexpression (Fig. 4g). In addition, another G4-stabilizing agent (TMPyP4) similarly reduced *TRA2B* promoter activity and its transcription (Supplementary Fig. S4). We also examined whether hnRNPU interacted with G4 in the promoter of *TRA2B*. As shown in Fig. 3d, ChIP assays showed that hnRNPU did not bind to the region containing G4.

**Figure 5:**
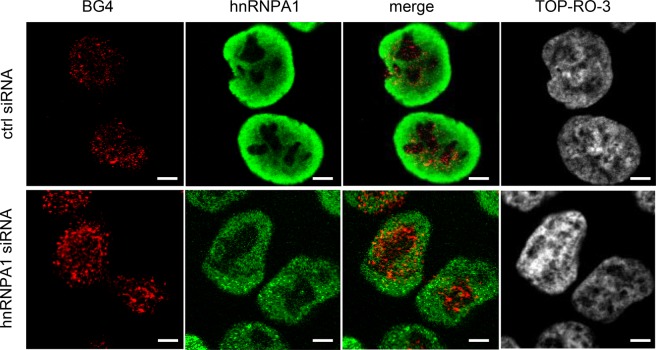
Effects of *hnRNPA1* knockdown on formation of G-quadruplex (G4) in HCT116 cells. After treatment of HCT116 cells with control or *hnRNPA1* siRNA for 48 h, G4 formation (red) and hnRNPA1 (green) were examined by immunofluorescence staining using anti-hnRNPA1 and BG4 antibodies. Nucleoli were counterstained with TO-PRO-3. Scale bars, 5 μm.

To further confirm the regulation of G4 structure formation by hnRNPA1, we performed double-immunofluorescence staining with anti-hnRNPA1 and BG4 antibodies. As shown in Fig. 5, confocal laser microscopy revealed that only small numbers of G4 structure were detected with BG4 antibody in control siRNA-treated HCT116 cells, whereas the numbers of G4 structure formation were substantially increased when hnRNPA1 was silenced.

These results suggest that hnRNPA1 may associate with the G4 sequence and interfere with G4 formation, allowing *TRA2B* transcription. The reduction of hnRNPA1 may facilitate G4 formation and downregulate *TRA2B* transcription.

### Regulation of *TRA2B* transcription by hnRNPU

Although we could not detect any interaction between hnRNPU and the G4 structure, hnRNPU knockdown decreased the luciferase reporter activity of the *TRA2B* promoter (−398 to +107 bp) by 50% (Fig. 3a and Supplementary Fig. S5a). To determine the hnRNPU-responsive region in the promoter, serially truncated segments of the promoter were prepared and used for the dual-luciferase reporter assay. As shown in Supplementary Fig. S5a, the hnRNPU-responsive site(s) seemed to lay between −231 and −89 bp. When hnRNPU was overexpressed, the activity of the −231 to +107 bp region was roughly doubled (Supplementary Fig. S5b). This functional promoter region was included in the primer for ChIP (−243 to −38) in Fig. 3d. However, ChIP assays with different sets of primers did not show any binding of hnRNPU to the region of −231 to −89 (Supplementary Fig. S5c), suggesting that hnRNPU might interact with the region via other proteins or RNAs to promote transcription.

### Expression of *hnRNPA1*, *hnRNPU*, *TRA2β1*, and *TRA2β4* in colon cancers and regulation of colon cancer cell growth by hnRNPA1 and hnRNPU

**Figure 6:**
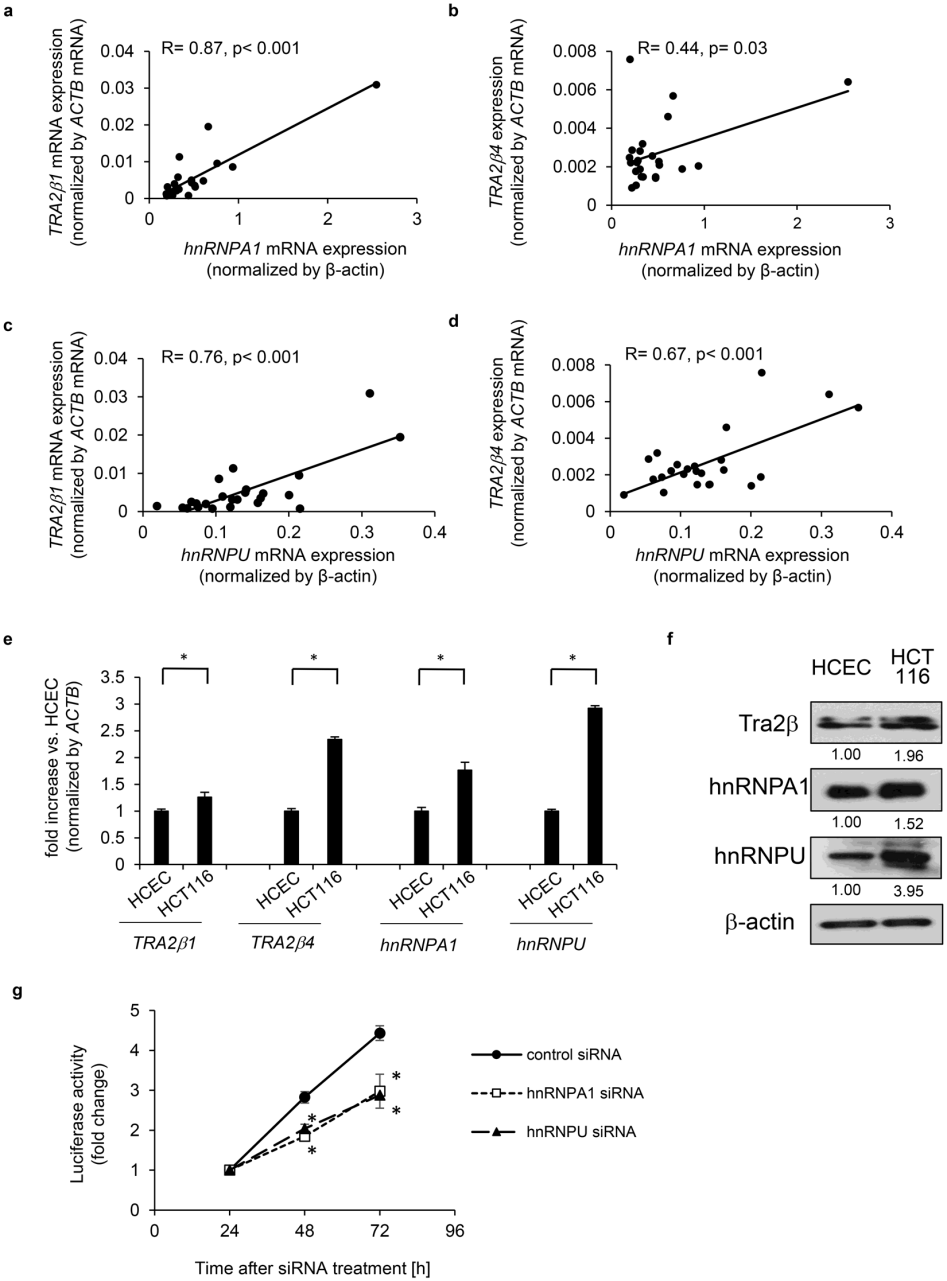
Relationship between *TRA2B* transcript levels and *hnRNPA1* or *hnRNPU* mRNA levels in colon cancer. (**a–d**) Amounts of *hnRNPA1*, *hnRNPU* or *TRA2β1* and *TRA2β4* were measured by RT-qPCR in 24 patients with colon cancer. *ACTB* mRNA was used as an internal control for normalization. The correlations between *hnRNPA1* and *TRA2β1* (**a**), *hnRNPA1* and *TRA2β4* (**b**), *hnRNPU* and *TRA2β1* **(c**), or *hnRNPU* and *TRA2β4* (**d**) were analyzed by Pearson’s correlation analysis. (**e**) Comparison of mRNA levels of *hnRNPA1*, *hnRNPU* or *TRA2β1* and *TRA2β4* in normal colon cell line (HCEC) and colon cancer cell line (HCT116). (**f**) Comparison of protein levels of Tra2β, hnRNPA1, and hnRNPU in HCEC and HCT116. (**g**) Effects of *hnRNPA1* or *hnRNPU* knockdown on growth of HCT116 cells. After treatment of HCT116 cells with control, *hnRNPA1*, or *hnRNPU* siRNA, growth of these cells was monitored by counting the number of cells using CellTiter-Glo Luminescent Cell Viability Assay (Promega). Values are means ± SD, n = 4. *Significantly different compared with control siRNA-treated cells (*p* < 0.05) by two-tailed Student’s *t*-test.

Using Tissue Scan RT Colon Cancer Disease Panels III HCRT103 (OriGene, MD, USA), mRNA levels of *hnRNPA1*, *hnRNPU*, or *TRA2β1* and *TRA2β4* levels in 24 patients with colon cancers were measured by RT-qPCR. As shown in Fig. 6a,b, *hnRNPA1* mRNA levels were positively correlated with the expression levels of *TRA2β1*, whereas expression levels of *hnRNPA1* showed weak correlation with those of *TRA2β4* in the colon cancers. *HnRNPU* mRNA levels were positively correlated to the expression levels of *TRA2β1* and *TRA2β4* (Fig. 6c,d). We further examined the expression levels of these mRNAs in normal colonic epithelial cells (HCEC-1CT) and colon cancer cells (HCT116). HCT116 cells expressed higher levels of *TRA2β1*, *TRA2β4*, *hnRNPU*, and *hnRNPA1* mRNAs than those in HCEC-1CT cells (Fig. 6e). In addition, HCT116 cells expressed significantly higher amounts of Tra2β, hnRNPA1, and hnRNPU proteins compared with HCEC-1CT cells (Fig. 6f). Although this is the limited survey, these results suggest that hnRNPA1 and hnRNPU may contribute Tra2β overexpression in colon cancer cells.

We also examined the effects of hnRNPA1 and hnRNPU on the growth of HCT116 cells. Cell growth assessed by the CellTiter-Glo Luminescent Cell Viability Assay (Promega, Madison, WI, USA) revealed that knockdown of hnRNPA1 or hnRNPU significantly decreased the growth rate of HCT116 cells (Fig. 6g).

## Discussion

In this study, we identified hnRNPA1 and hnRNAU as *TRA2β4* exon 2-binding proteins. Our data demonstrated that these 2 hnRNPs regulated alternative splicing of exon 2. The minigene assay indicated that hnRNPA1 facilitated inclusion of exon 2, whereas hnRNPU promoted skipping of the exon. In addition to regulating the splicing reaction, we found that hnRNPA1 and hnRNAU could upregulate *TRA2B* transcription in different ways.

HnRNPA1 and hnRNPU are multifunctional proteins that have both DNA- and RNA-binding abilities. These 2 hnRNPs are splicing factors as well as transcription-regulating factors. HnRNPA1 regulates the transcription of genes in a variety of ways. It has been shown that hnRNPA1 interacts with the promoter region of the genes for thymidine kinase^32^, γ-fibrinogen^33^ chain and *APOE*^34^, and activates transcription of these genes in immortalized or cancer cell lines. The reduction of hnRNPA1 likely changed alternative splicing of *TRA2B* exon 2. However, it decreased expression levels of both *TRA2β1* and *TRA2β4*. In fact, the reduction of hnRNPA1 significantly decreased the promoter activity of *TRA2B*.

It was recently shown that hnRNPA1 regulates the stability of G-quadruplex (G4) structures in certain promoters and regulates the expression of cancer-related genes, such as *KRAS*^35,36^. G4 is now recognized as a crucial regulator of cancer-related genes^26^ and is considered a potential target for cancer treatment^37^. Paramasivam *et al*. reported that hnRNPA1 unwound G4 and promoted *KRAS* gene expression^36^. Several studies suggested that the interaction between hnRNPA1 and G4 in telomeric repeat-containing RNA (*TERRA*) was important to maintain telomeres. HnRNPA1 associated with *TERRA* and altered the binding of protection of telomeres 1 (POT1) to telomeric single-stranded DNA, resulting in the prevention of *TERRA*-mediated telomerase inhibition^38,39^. Liu *et al*. showed that telomere RNA G-quadruplex played a crucial role in the association with hnRNPA1 *in vitro* and in living cells^40^. These results indicated that G4-dependent binding of hnRNPA1 to telomere RNA might regulate telomere extension by mediating telomerase activity. Based on these findings, we were particularly interested in the presence of the putative G4 sequences (5′-GGGGTGGGGCCCTGCGAGGGCGGG-3′) in the *TRA2B* gene promoter region (−214 to −188 bp). This sequence structure displayed a G4-specific spectrum (the highest peak at 265 nm and the lowest peak at 240 nm) by circular dichroism analysis. The presence of a G4 structure was confirmed by EMSA with a G4-specific antibody (BG4) as well as a ChIP-PCR assay. However, further experiments are needed to characterize structure and dynamics of G4 in the *TRA2B* promoter in colon cancer cells. Our results suggest that hnRNPA1 may associate with the G4 sequence and modify the G4 structure to promote *TRA2B* gene transcription. Notably, treatment with a G4 stabilizer, pyridostatin (PDS), decreased the *TRA2B* transcript levels in association with the decrease in the *TRA2B* promoter activity. Furthermore, overexpression of hnRNPA1 significantly increased both *TRA2β1* and *TRA2β4* levels, whereas the increases were completely cancelled with PDS. Thus, our results suggest that hnRNPA1 may regulate *TRA2B* transcription mainly through the G4 structure. Consistent with these findings, the results of immunofluorescence staining with an anti-G4 antibody (BG4) showed that G4 was masked and only small amounts of G4 formation were observed under ordinary conditions. However, G4 structure formation was likely facilitated once hnRNPA1 was silenced. These data are consistent with several reports that have shown that hnRNPA1 could un-wind G4 structure formation in telomeres^41–43^.

HnRNPU also binds to promoters and regulates the transcription of several genes such as *Apod*^44^, *Klf2*^45^, *Oct4*^46^, and *Opn*^47^ in murine cells including fibroblast cells, ES cells and macrophage cells. Furthermore, Nozawa *et al*. reported that hnRNPU interacted with chromatin and changed its structure via chromatin-associated RNAs as a function of transcription^48^. In addition, knockdown of hnRNPU reportedly reduced global transcription levels after 72 h, but not after 48 h^46^. In our experimental conditions, we examined gene expression 48 h after treatment with hnRNPU siRNAs. Although the *TRA2B* promoter possessed an hnRNPU-responsive region, we could not detect any direct association of hnRNPU with the promoter. In addition, it did not interact with G4 in the *TRA2B* promoter. Thus, it is possible that hnRNPU functionally regulates the promoter by interacting with certain RNAs or proteins. Past studies reported that hnRNPU formed a protein complex with RNA polymerase II at the promoter regions to promote transcription^46,49,50^. The same mechanism could exist in the *TRA2B* promoter.

It is known that several hnRNP family proteins interact together at alternative splice sites. Moreover, the hnRNP family regulates both inclusion and skipping of exons depending on the type of genes^51^. According to our results, hnRNPA1 and hnRNPU promoted and decreased the splicing *TRA2β* pre-mRNA. However, both *TRA2β1* and *TRA2β4* were decreased after knockdown of either hnRNPA1 or hnRNPU. Thus, hnRNPA1and hnRNPU mainly regulate *TRA2B* transcription rather than *TRA2β* pre-mRNA processing.

Tra2β, *TRA2β4*, hnRNPA1 and hnRNPU were reportedly significantly associated with accelerated cell growth. Knockdown of hnRNPA1 induced cell cycle arrest in oral squamous cancer cells and lung adenocarcinoma^29,31^. Knockdown of hnRNPU induced cell senescence and increased CDKN1A^30^. Notably, we also reported that *TRA2β4* knockdown stimulated *CDKN1A* transcription and increased p21 levels in colon cancer cells, resulting in cell senescence^12^. *TRA2β4* interacted with Sp1 through a Sp1-binding sequence (485-GGGG-488) in a stem-loop structure of exon 2^12^. *TRA2β4* associated with nucleolin and was preferentially localized in the nuclei of human colon cancer cells^15^. Overexpression of *TRA2β4* significantly decreased *CDKN1A* mRNA levels and accelerated cell growth^12^. As to Tra2β, this protein could bind to the BCL2 3′-UTR and disrupt miR-204-binding to the BCL2 3′-UTR. Overexpressed Tra2β could increase Bcl-2 expression through competition with miR-204, facilitating colon cancer cell growth^16^. Thus, ectopic overexpression of Tra2β protein and *TRA2β4* RNA causes abnormal growth of colon cancer cells. A pathological study showed that hnRNPA1 and hnRNPU were expressed significantly higher in the nuclei of colorectal cancer tissue than normal colon tissue^52^. Considering these results, *TRA2B* gene transcripts and hnRNPA1 or hnRNPU may be closely interacting regulators of the cell cycle or cell growth, which is a crucial phonotype of cancer cells. The mechanisms of regulation of cell cycle by the hnRNP family are not fully elucidated. However, the mechanism may involve *TRA2B* products in the downstream pathway.

In summary, we identified hnRNPA1 and hnRNPU as *TRA2β4* exon 2-binding proteins. These two hnRNPs regulated *TRA2B* transcription and alternative splicing of the exon 2. HnRNPA1, but not hnRNPU, interacted with a G4 sequence in the *TRA2B* promoter and facilitated *TRA2B* transcription. Tra2β protein and *TRA2β4* play crucial roles in abnormal colon cancer cell growth, and G4 is now recognized as a crucial regulator for cancer-related genes. These results suggest that hnRNPA1/G4 may participate in ectopic overexpression of *TRA2B* products in colon cancer cells.

## Materials and Methods

### Cell culture and knockdown experiments

Human HCT116 colon cancer cells were cultured in Dulbecco’s modified Eagle’s medium (DMEM; Nacalai Tesque, Tokyo, Japan) supplemented with 5% heat-inactivated fetal bovine serum (FBS) at 37 °C in 5% CO_2_. Human colon epithelial cells (HCEC-1CT) were obtained from EVERCYTE (Vienna, Austria) and cultured using DMEM/Medium 199 Earle’s. Cell growth was assessed using CellTiter-Glo Luminescent Cell Viability Assay (Promega) according to the manufacturer’s instructions.

*HnRNPA1* siRNA#1 and siRNA#2 were obtained from Cell Signaling Technology (#7668; Danvers, MA, USA) and Qiagen (SI00300419; Hilden, Germany), respectively. *HnRNPU* siRNA#1 (sc-38298; Santa Cruz Biotechnology, Santa Cruz, CA, USA) and siRNA#2 (SI02781002; Qiagen) were also purchased. HCT116 cells were treated with 20 nM negative control siRNA (AllStars Negative; Qiagen), hnRNPA1 or hnRNPU siRNAs using Lipofectamine RNAiMAX Transfection Reagent (Thermo Fisher Scientific, Waltham, MA, USA) for 48 h.

### Western blot analysis

Whole cell lysates were prepared using RIPA buffer (10 mM Tris-HCl, pH 7.4; 1% Nonidet P-40; 1 mM EDTA; 0.1% SDS; 150 mM NaCl) containing a complete protease inhibitor cocktail and a phosphatase inhibitor cocktail (Roche Diagnostics, Basal, Switzerland). The extracted proteins (10 μg protein) were separated by SDS-PAGE and then transferred to a polyvinylidene fluoride membrane (Bio-Rad, Hercules, CA, USA). The membranes were incubated with a mouse monoclonal anti-GAPDH antibody (Santa Cruz Biotechnology), anti-hnRNPA1 antibody (Santa Cruz Biotechnology), anti-hnRNPU antibody (Bethyl Laboratories, TX, USA), or anti-Tra2β antibody (Abcam, Cambridge, UK) at 1:1000 dilution. The bound antibodies were detected with Pierce Western Blotting Substrate (Thermo Fisher Scientific).

### Real-time quantitative PCR (RT-qPCR)

Total RNAs were extracted from HCT 116 cells using RNAiso Plus (Takara, Otsu, Japan). Isolated RNA (1 μg) was reverse-transcribed using a PrimeScript RT Master Mix (Takara). RT-qPCR was conducted using SYBR Green Master Mix (Applied Biosystems, Foster City, CA, USA). The following primer sets were used; 5′-CGGCGAGCGGGAATCCCG-3′ (forward) and 5′-GACATGGGAGAATGGCTGTGGC-3′ (reverse) for *TRA2β1*, 5′-AGGAAAATGCGGAAGTCGTC-3′ (forward) and 5′-GCGTAGTGCTTTCTGATTCCAG-3′ (reverse) for *TRA2β4*, 5′-AAGCAATTTTGGAGGTGGTG-3′ (forward) and 5′-ATAGCCACCTTGGTTTCGTG-3′ (reverse) for *hnRNPA1* mRNA, and 5′-AACAGAGGTGGTGGCCATAG-3′ (forward) and 5′-GTAACTACCACGGCCAGGAA-3′ (reverse) for *hnRNPU* mRNA. Glyceraldehyde-3-phosphate dehydrogenase (*GAPDH*) mRNA levels were measured as an internal control for normalization. The following primer set was used:; 5′-TGCACCACCAACTGCTTAGC-3′ (forward) and 5′-GGCATGGACTGTGGTCATGAG-3′ (reverse). Levels of mRNAs were measured by the comparative ΔΔCt method using *GAPDH* mRNA and expressed as values relative to the normal samples.

### Overexpression of hnRNPA1 and hnRNPU

Full-length *hnRNPA1* and *hnRNPU* mRNAs were cloned into the pEBMulti-Bsd vector (Fujifilm Wako Pure Chemical, Osaka, Japan). For full-length *HNRNPA1*, cDNA was amplified by PCR using the following primer set: 5′-GGATCCGCCGTCATGTCTAAGTCAGAGTCT-3′ (forward) and 5′-GCGGCCGCTTAAAATCTTCTGCCACTGCCATA-3′ (reverse). A full-length *hnRNPA1* was amplified using the following primer set; 5′-GGATCCCTCACCATGAGTTCCTCGCCTGTT-3′ (forward) and 5′-GCGGCCGCTCAATAATATCCTTGGTGATAATG-3′ (reverse). The amplified products were subcloned using BamHI and NotI restriction sites (these sequences are underlined). HCT116 cells were transfected with these constructs for 48 h at a final concentration of 1.5 μg/mL using Lipofectamine 2000 reagent (Thermo Fisher Scientific).

### Biotinylated RNA Pull-Down assay

The assays were performed by the method as previously described^15^. Briefly, for each target mRNA fragment, template DNA was synthesized by PCR with gene specific primer sets containing T7 RNA polymerase promoter sequence. Biotinylated RNA fragments were generated by *in vitro* transcription using MAXIscript T7 Transcription Kit (Thermo Fisher Scientific) and Biotin-11-CTP (PerkinElmer, Waltham, CT, USA). After incubation of 2.5 μg biotinylated RNA with 80 μg of whole cell lysates in TENT buffer (10 mM Tris-HCl buffer, pH 8.0, 250 mM NaCl, 0.5% Triton X-100, and 1 mM EDTA) for 30 min, Dynabeads M-280 Streptavidin (Thermo Fisher Scientific) were added and incubated at room temperature for 1 h. The proteins bound to the beads were isolated with magnetic beads separator and followed by western blot assay or liquid chromatography-mass spectrometry assay (nanoLC-MS/MS, CapLC, Q-Tof).

### Ribonucleoprotein immunoprecipitation (RIP) analysis

The assays were performed using the method as previously described^15^. Briefly, 20 μl of protein A Sepharose (Sigma-Aldrich) and 3 μg of anti-hnRNPU or anti-hnRNPA1 antibody were mixed in 1 mL of NT-2 buffer (50 mM Tris HCl, pH 7.4; 150 mM NaCl; 1 mM MgCl_2_; 0.05% (v/v) Nonidet P-40). Cells were lysed in lysis buffer (25 mM Tris-HCl pH 7.5, 150 mM NaCl, 1 mM EDTA, 1% (v/v) Nonidet P-40, 5% (v/v) glycerol, and 100 U/mL RNase inhibitor (Nacalai Tesque). The lysates were mixed with protein A Sepharose, for 2 h with rotation. Subsequently, those mixtures were treated with TURBO DNase (Thermo Fisher Scientific) for 10 min at 37 °C. Bound RNAs were extracted using RNAiso plus and then subjected to RT-qPCR.

### Promoter activity assay

The 5′-flank of the human *TRA2B* gene was cloned into the pGL3-basic luciferase reporter vector (Promega). The first PCR was performed using genomic DNA from HCT116 cells as a template. The *TRA2B* proximal promoter region (from −398 to +107 bp) was amplified using the following primer set: 5′-AATAAGGCGGAGAAAGGAATTGAGATTGGGGGAA-3′ (forward) and 5′-GACTCCTGGCTGCTGTCGCCGGTCGAT-3′ (reverse). The amplified products were subcloned into the pGL3-basic vector using SacI and Xho1 restriction sites. After treatment with control or hnRNPU siRNA for 24 h, pGL-3 luciferase constructs (200 ng) were co-transfected with pRL-CMV vector (100 ng) using Lipofectamine 2000 (Thermo Fisher Scientific) for 24 h. These cells were harvested, and firefly and *Renilla* luciferase activities were measured by luminometer using the Dual-Luciferase Reporter Assay System (Promega).

### Minigene assay

To assess the alternative splicing of *TRA2B* pre-mRNA, genomic sequences of exon 1 to exon 3 were cloned as a minigene and inserted into the multi-cloning site of pcDNA3.1 using restriction enzymes of XhoI and BamHI as previously reported^53^.

After HCT116 cells were treated with control, *hnRNPU* or *hnRNPA1* siRNA for 24 h, 2 μg of pcDNA3.1 with minigene or mock vector was transfected for 24 h and then total RNAs were extracted using an RNeasy mini kit (Qiagen). Complementary DNA was obtained by reverse transcription using SuperScriptIII (Thermo Fisher Scientific) and gene-specific primers (5′-TATCCGAGGGTTCAACCTCGA-3′). PCR was performed for the minigene-originated complementary DNA with the following primer set: 5′-GGGGATCCGACCGGCGCGTCGTGCGGGGCT-3′ (forward) and 5′-GGGCTCGAGTACCCGATTCCCAACATGACG-3′ (reverse). PCR products were separated by 1.5% agarose gel electrophoresis. Amounts of each band in the gel were quantified and analyzed by Image J software (NIH).

### Chromatin immunoprecipitation (ChIP) assay

ChIP assays were conducted using the EZ-Magna ChIP A (Millipore, MA, USA). HCT116 cells in 100-mm dishes at 80–90% confluence (approximately 2 × 10^8^ cells) were fixed in 10 mL DMEM containing 1% formaldehyde for 10 min at room temperature. Cells were washed with ice-cold PBS containing a protease inhibitor cocktail, and the chromatin was isolated according to the manufacturer’s instruction. Isolated chromatin was broken into 200–500 bp sizes using a sonicator (Digital Sonifire; BRANSON, Danbury, CT, USA). Chromatin suspensions (500 μL) were sonicated for 4 cycles (10 s on/60 s off) with a 20% amplitude. After centrifuging the samples at 800 × *g* for 5 min at 4 °C, the supernatants were collected. Fragmented chromatin was diluted in 10 × ChIP dilution buffer and incubated overnight with one of the following: 5 μg of anti-hnRNPA1 antibody, normal mouse IgG, anti-hnRNPU antibody, normal rabbit IgG, BG4 antibody or normal goat IgG. After bound DNAs were immunoprecipitated with magnetic beads, the G4-containing region was amplified by PCR using the following primer set: 5′-TAGCTTTCCCGCCTCATAGA-3′ (forward) and 5′-CGCACGGGCTCTAACTCTAC-3′ (reverse).

### Circular dichroism (CD) measurement

CD spectra were measured using the J-820 spectropolarimeter (JASCO, Tokyo, Japan) with a 1-mm path length quartz cuvette. The single strand DNA sequences of G4 (5′-GGGGTGGGGCCCTGCGAGGGCGGG-3′) and the sequence with mutation (5′-GGAGTGGAGCCCTGCGAGAGCGAG-3′) were purchased from Fasmac Co. (Kanagawa, Japan). Single strand DNAs at a concentration of 10 μM were dissolved in 50 mM Tris-HCl (pH 7.4) containing 100 mM KCl, heated at 95 °C for 5 min and then slowly cooled to 25 °C. CD spectra from 220 to 320 nm were scanned at 25 °C as follows: 100 nm/min scanning speed, 1-nm band width, 0.5-nm data interval and 1-s response.

### Electrophoretic mobility shift assay (EMSA)

An IRDye700-labeled G4 sequence of the *TRA2B* promoter (5′-TGGGGGTGGGGCCCTGCGAGGGGCGGGGT-3′) was obtained from IL-COR Bioscience (Lincoln, NE, USA). The labeled oligonucleotides were dissolved in 10 mM Tris-HCl buffer (pH 7.4) containing 100 mM KCl, heated at 95 °C for 5 min and then slowly cooled to 25 °C. The labeled oligonucleotides were incubated with BG4 antibody (Absolute Antibody; Oxford, Oxfordshire, UK) in 20 mM HEPES buffer (pH 7.5) containing 100 mM KCl, 0.01% (v/v) NP40, 5% (v/v) glycerol, and 5 mM MgCl_2_ for 1 h. Human recombinant hnRNPA1 protein was purchased from ATGen (Gyeonggi, South Korea). The complexes were resolved on a 4% polyacrylamide gel and imaged by ODYSSEY (LI-COR Bioscience).

### Immunofluorescence staining

After HCT116 cells were treated with control or hnRNPA1 siRNA for 48 h, they were fixed with 4% paraformaldehyde phosphate buffer solution (Nacalai Tesque, Kyoto, Japan) and treated with 0.5% (v/v) Triton X. These cells were further treated with 200 μg/mL RNase A for 1 h at 37 °C and then incubated overnight with 0.4 ng/μL of hnRNPA1 antibody or 4 ng/μL of BG4 antibody at 4 °C. After washing with PBS, the signals were visualized with Alexa Fluor 488 anti-mouse IgG or Alexa Fluor 555 anti-goat IgG (Thermo Fisher Scientific). The nuclei were stained with TO-PRO-3 (Thermo Fisher Scientific) using a coverslip with Vectashield (Thermo Fisher Scientific).

## Supplementary information

Supplementary Information

## Data Availability

All data generated or analysed during this study are included in this published article (and its Supplementary Information files).
