# Supplementary material for: HnRNPA1 interacts with G-quadruplex in the TRA2B promoter and stimulates its transcription in human colon cancer cells
Source: Sci Rep. 2019 Jul 16;9:10276. doi: 10.1038/s41598-019-46659-x (PMC6635519; doi:10.1038/s41598-019-46659-x)
Supplement: Supplementary file 1 — Supplementary Information [file 41598_2019_46659_MOESM1_ESM.pdf]

HnRNPA1 interacts with G-quadruplex in the *TRA2B* promoter and stimulates its transcription in human colon cancer cells

Tatsuya Nishikawa[1,*], Yuki Kuwano[1], Yumiko Takahara[2], Kensei Nishida[1], and Kazuhito Rokutan[1]

1 Department of Pathophysiology, Institute of Biomedical Sciences, Tokushima University Graduate School, Tokushima 770–8503, Japan

2 Student Lab, Tokushima University Faculty of Medicine, Tokushima, Japan.

* nishikawa.tatsuya@tokushima-u.ac.jp

Supplementary Figures

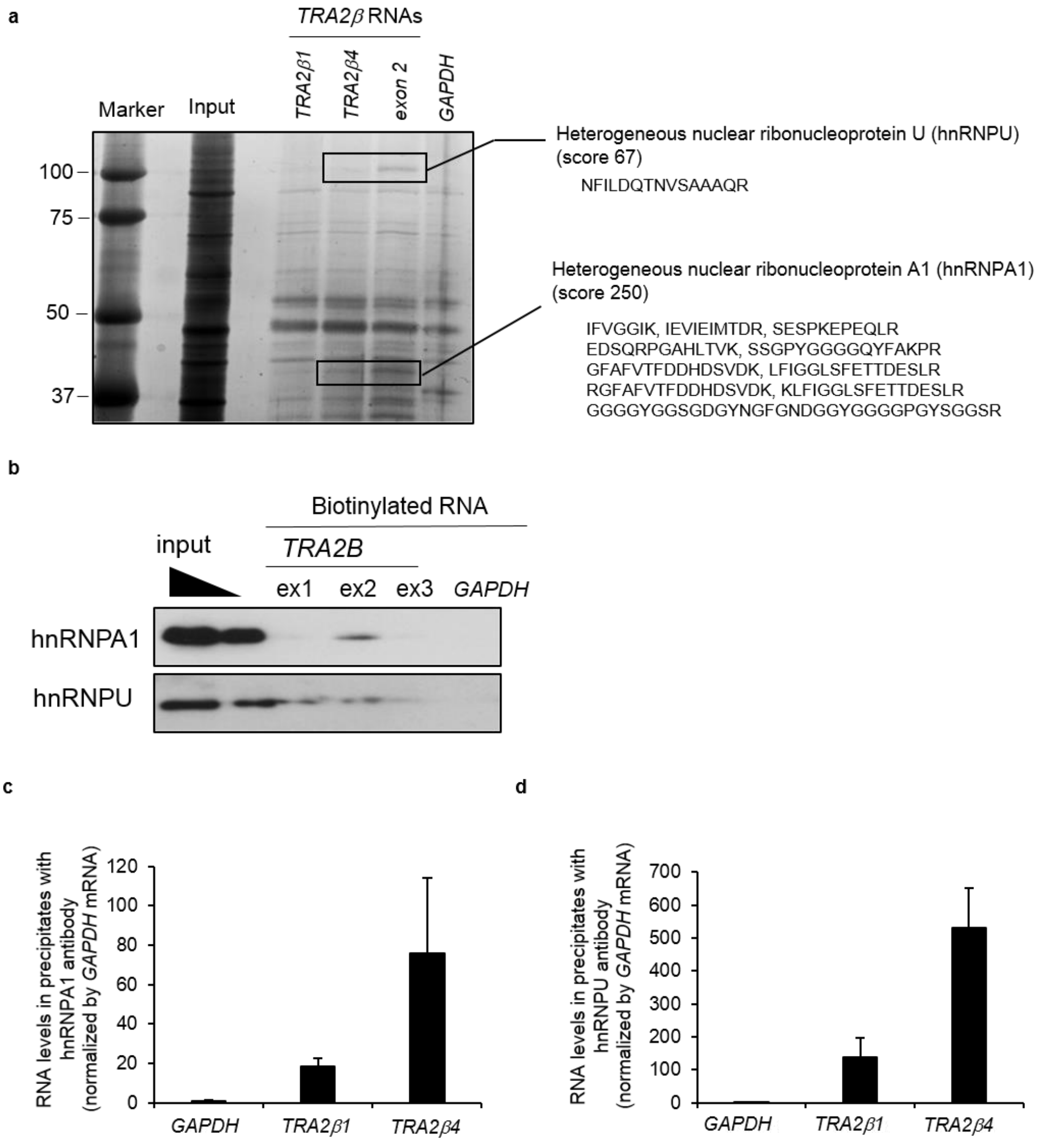

Supplementary Figure S1. Identification of *TRA2β4*-binding proteins.

(a) After biotin pull-down assay using the biotinylated probes, the purified proteins were resolved by SDS-PAGE and visualized by silver staining. LC-MS/MS identified hnRNPU as one of the major ~100 kDa proteins in a previous report[15]. LC-MS/MS of the ~40 kDa proteins identified hnRNPA1 as one of the predominant proteins in the precipitate. (b) After biotin pull-down assay, bound proteins were detected by western blotting using anti-hnRNPA1 and anti-hnRNPU antibodies. (c) and (d) After RNA immunoprecipitation with an anti-hnRNPA1 or anti-hnRNPU antibody, precipitated *TRA2β1* and *TRA2β4* were measured by RT-qPCR using *GAPDH* mRNA as an internal control for normalization. Values are means ± SD, n = 3.

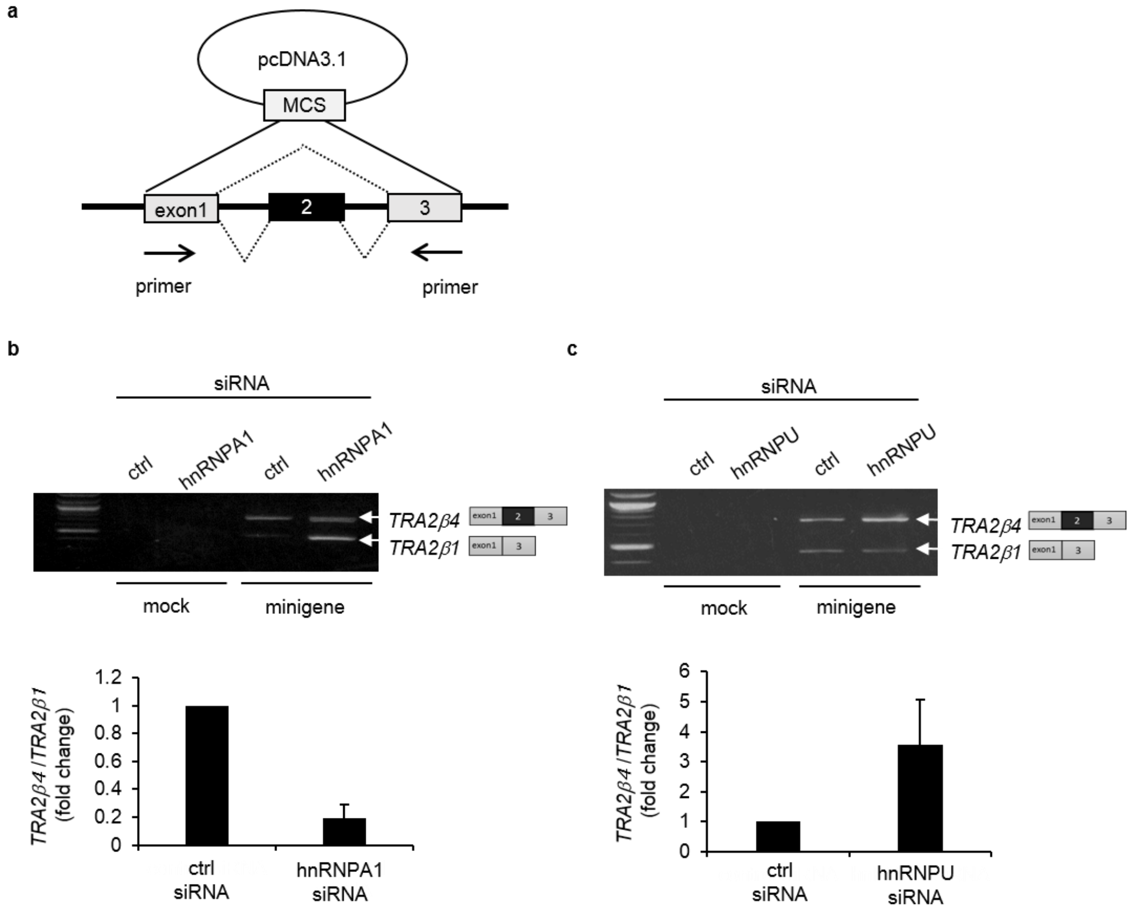

Supplementary Figure S2. Effects of treatment with *hnRNPA1* or *hnRNPU* siRNA on alternative splicing of *TRA2β* pre-mRNA.

(a) A minigene, which contained *TRA2B* exons 1 to 3, was inserted into a multi-cloning site (MCS) in a pcDNA3.1 vector. (b) and (c) After treatment of HCT116 cells with control, *hnRNPA1*, or *hnRNPU* siRNA, the minigene or mock structure was transfected into these cells. The minigene transcripts were amplified by PCR using the primer set indicated by arrows in Fig. 4a, and amplified products were separated by agarose gel electrophoresis. The upper bands indicated inclusion of exon 2 (*TRA2β4*) and the lower band indicated skipping of exon 2 (*TRA2β1*). The intensity of each band in the gel was quantified and analyzed by Image J software (NIH) in 3 independent experiments. The intensities were normalized by those observed in control siRNA-treated cells. The *TRA2β1*/*TRA2β4* ratio was calculated. Values are means ± SD, n = 4.

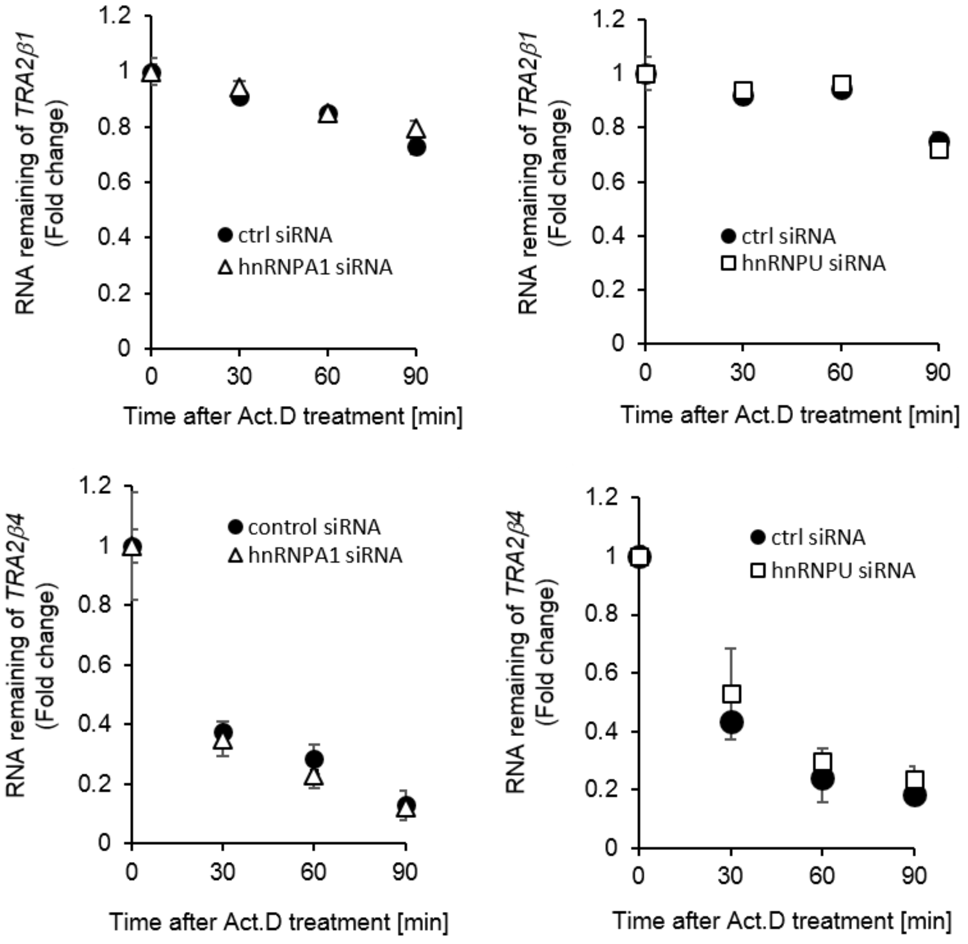

Supplementary Figure S3. Effects of *hnRNPA1* and *hnRNPU* on stability of *TRA2β1* and *TRA2β4.*

After HCT116 cells were transfected with the indicated siRNAs for 48 h, they were incubated in the presence of 2 μg/mL actinomycin D for the indicated times. *TRA2β1* and *TRA2β4* levels were measured by RT-qPCR and plotted on a logarithmic scale to calculate the time required for each RNA to reach one-half of its initial abundance. Values are means ± SD, n = 3. *, significantly different, compared with control siRNA ($p < 0.05$ by Student's *t*-test).

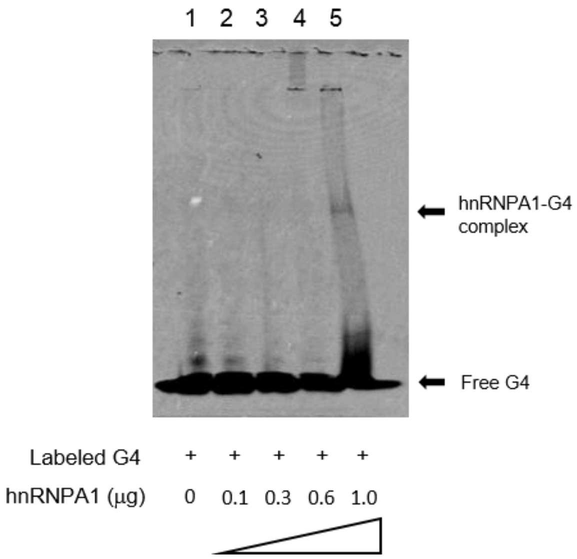

Supplementary Figure S4. Direct interaction between hnRNPA1 and G4 in the *TRA2B* promoter.

EMSA was performed as described in Materials and Methods. In this figure, addition of purified hnRNPA1 protein could form hnRNPA1-G4 complex.

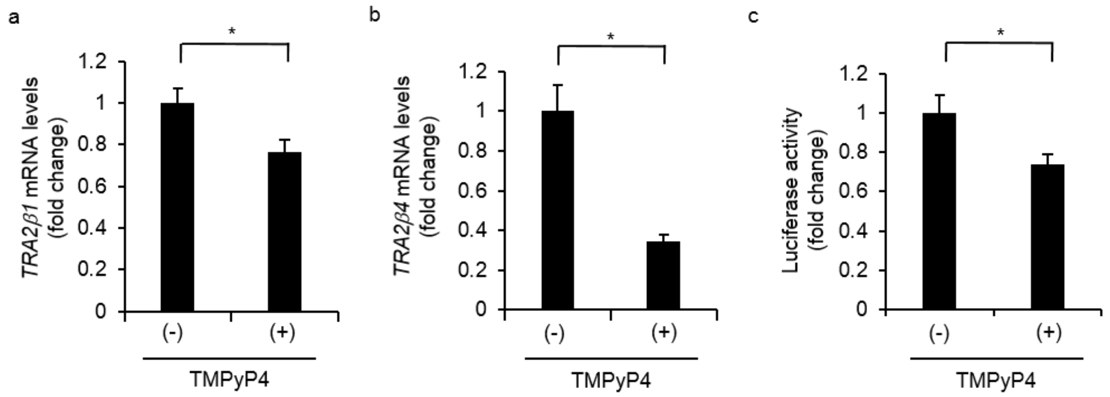

Supplementary Figure S5. Effects of a G4 stabilizer (TMPyP4) on *TRA2B* transcription.

(a) After HCT116 cells were treated with 100 μM TMPyP4 for 24 h, total RNA was extracted. The levels of *TRA2β1* and *TRA2β4* were measured by RT-qPCR. The values were normalized to those of *GAPDH* mRNA. (b) The pGL3 luciferase construct encoding the *TRA2B* proximal promoter (from -398 to +107 bp) was co-transfected with pRL-CMV vector for 24 h. After these cells were treated with or without 100 μM TMPyP4 for 24 h, dual-luciferase reporter assays were employed. Values (fold-changes) are means ± SD, n = 3. *, significantly different, compared with control ($p < 0.05$ by Student's *t*-test).

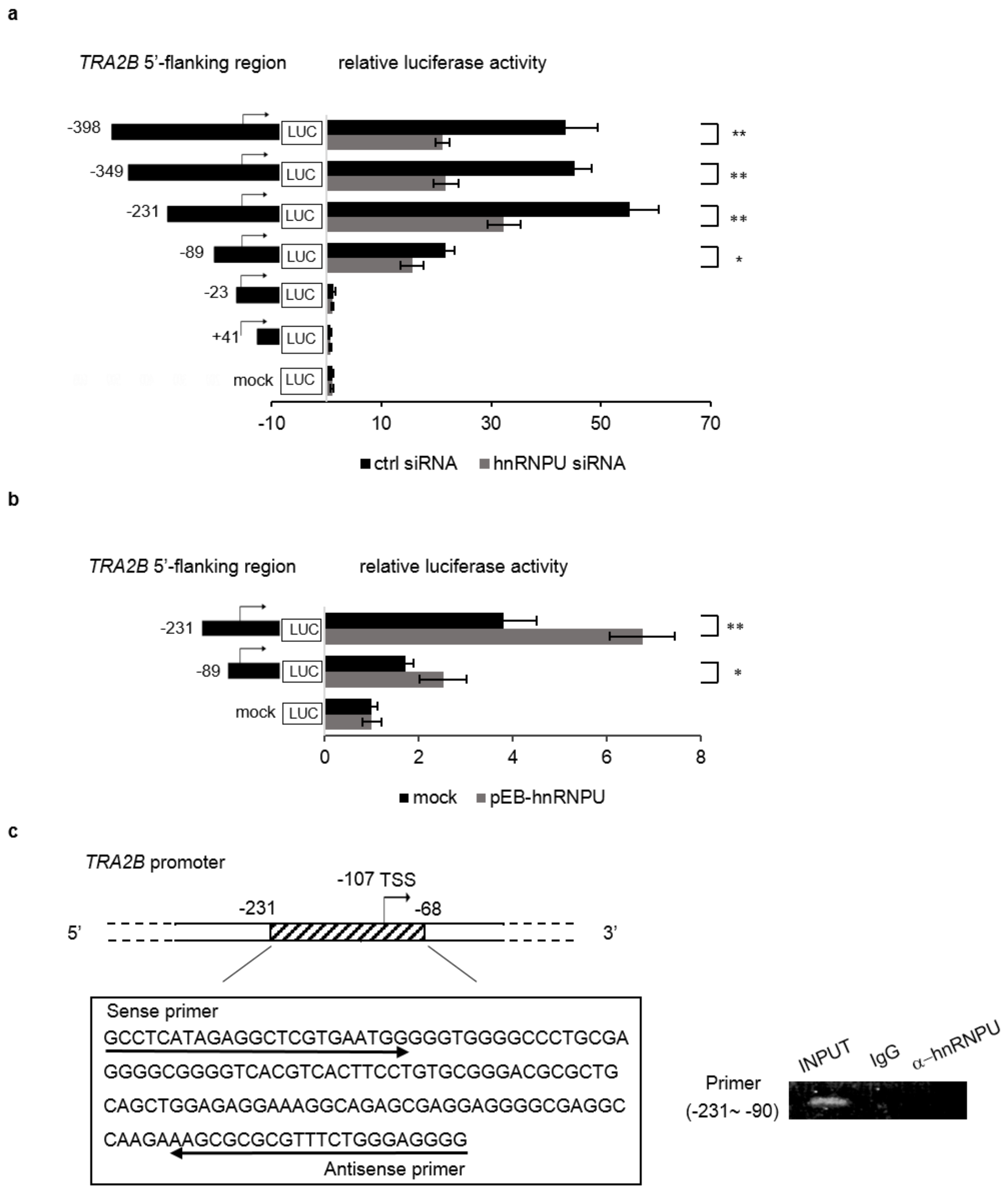

Supplementary Figure S6. Effects of hnRNPU on the *TRA2B* gene transcription.

(a) After treatment of HCT116 cells with control or *hnRNPU* siRNA for 24 h, the pGL3 luciferase construct with the *TRA2B* proximal promoter (-398 to +107 bp) or truncated promoter regions (-349 to +107 bp, -231 to +107 bp, -89 to 107bp, -23 to +107, or +41 to +107 bp) was co-transfected with pRL-CMV vector for 24 h and then dual-luciferase reporter assays were employed. Using a KOD-Plus-mutagenesis kit (TOYOBO, Osaka,

Japan), the pGL3 system containing -398 to +107 was used as a template to generate the deletion constructs using the same reverse primer and one of the following forward primers: 5’-ACACTCGGCAGGTTCCCTGG-3’ for -349 to +107 bp; 5’-GCCTCATAGAGGCTCGTGAATG-3’ for -231 to +107 bp; 5’-AGGAGCCTGGCTAAGGAGCGCC-3’ for -89 to +107 bp; 5’-AGCATTTCGGCTCTGAGCGGCTG-3’ for -23 to +107 bp; and, 5’-TAAGGAAGGTGCAAGAGGTTGGCAGCT-3’ for +41 to +276. (b) The promoter activity in hnRNPU-overexpressing cells was assessed by dual-luciferase reporter assays after transfection with the indicated constructs for 24 h. *, significant differences compared with control siRNA or mock ($p < 0.05$) by Student's t-test (n = 4). **, significant differences compared with control siRNA or mock ($p < 0.001$) by Student's *t*-test (n>3). (c) The promoter sequence of hnRNPU-responsive region (-231 to -68) is shown. ChIP-PCR was demonstrated using the set of indicated primers.

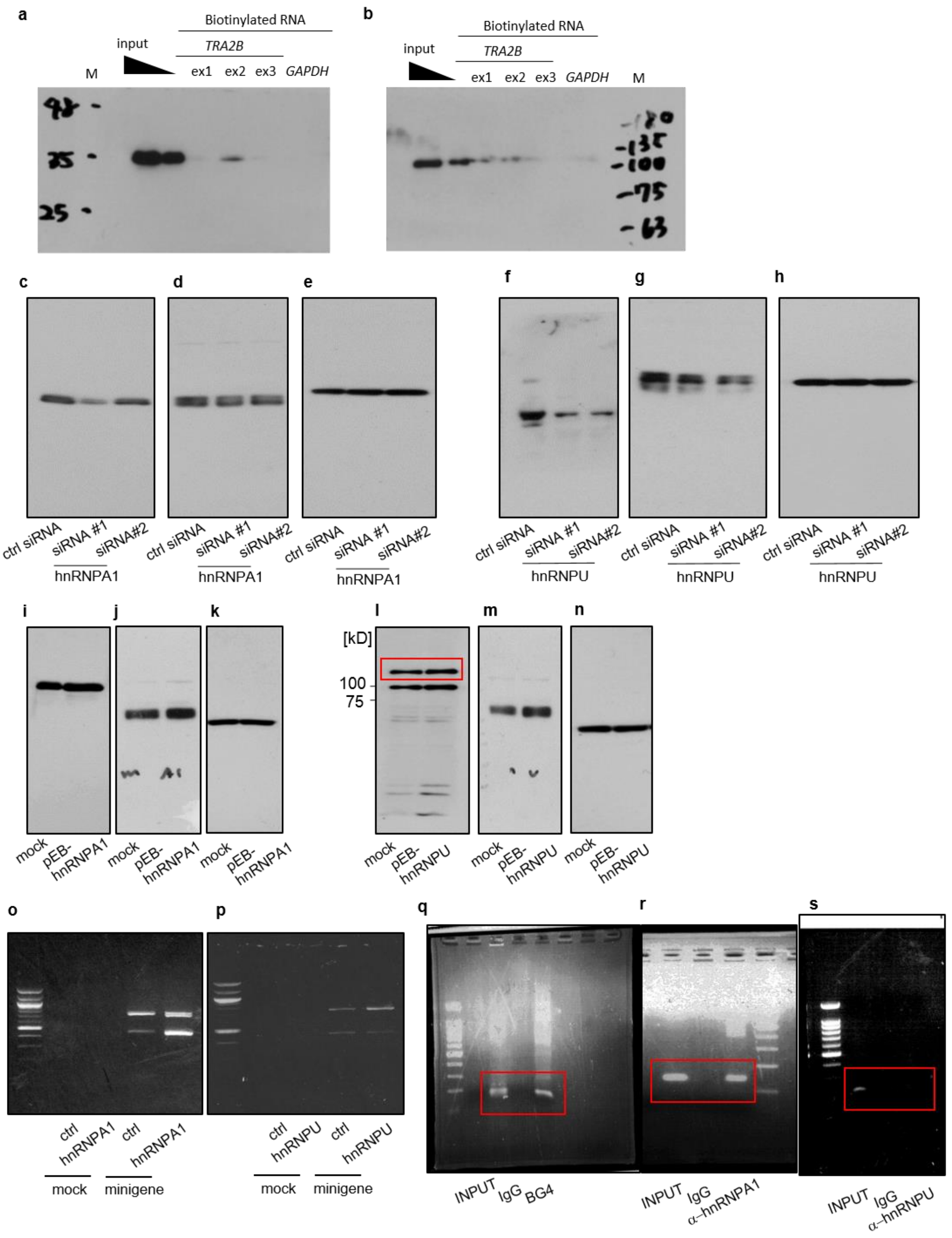
a
Biotinylated RNA
input
TRA2B
M
ex1 ex2 ex3 GAPDH
48
35
25
b
Biotinylated RNA
input
TRA2B
ex1 ex2 ex3 GAPDH M
180
135
100
75
63
c
d
e
f
g
h
ctrl siRNA siRNA #1 siRNA#2
hnRNPA1
ctrl siRNA siRNA #1 siRNA#2
hnRNPA1
ctrl siRNA siRNA #1 siRNA#2
hnRNPA1
ctrl siRNA siRNA #1 siRNA#2
hnRNPU
ctrl siRNA siRNA #1 siRNA#2
hnRNPU
ctrl siRNA siRNA #1 siRNA#2
hnRNPU
i
j
k
l
m
n
[kD]
100
75
mock pEB-hnRNPA1
mock pEB-hnRNPA1
mock pEB-hnRNPA1
mock pEB-hnRNPU
mock pEB-hnRNPU
mock pEB-hnRNPU
o
p
q
r
s
ctrl hnRNPA1 ctrl hnRNPA1
mock minigene
ctrl hnRNPU ctrl hnRNPU
mock minigene
INPUT IgG BG4
INPUT IgG α-hnRNPA1
INPUT IgG α-hnRNPU

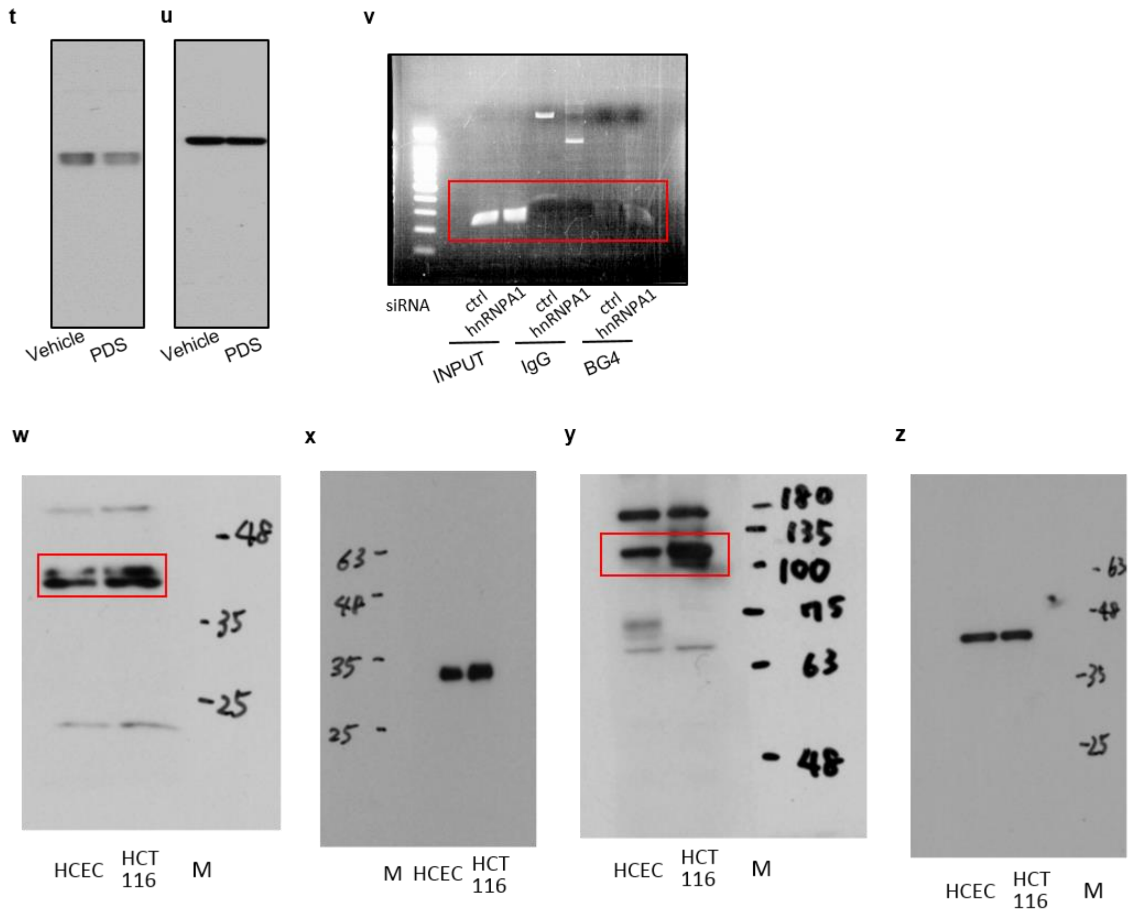

Supplementary Figure S7. Full-length blots of main figures.

The paired figures were as follows; (a)-(b): Supplementary Figure S1b; (c)-(e): Figure 1h; (f)-(h): Figure 1i; (i)-(k): Figure 2g; (l)-(n): Figure 2h; (o)-(p): Supplementary Figure S2b and S2c; (q)-(s): Figure 3d; (t)-(u): Figure 4c; (v): Figure 3g; (w)-(z): Figure 6f. The target bands used in the figure was indicated by red square.
